# The birth of neurotrauma: a historical perspective from the Academy of Multidisciplinary Neurotraumatology (AMN)

**DOI:** 10.25122/jml-2021-1006

**Published:** 2021

**Authors:** Dafin Muresanu, Stefana-Andrada Dobran, Dragos Cretoiu

**Affiliations:** 1. Department of Neuroscience, Iuliu Hatieganu University of Medicine and Pharmacy, Cluj-Napoca, Romania; 2. RoNeuro Institute for Neurological Research and Diagnostic, Cluj-Napoca, Romania; 3. Department of Cell and Molecular Biology and Histology, Carol Davila University of Medicine and Pharmacy, Bucharest, Romania

The study of neurotraumatology throughout the ages has shaped neuroscience, medicine, forensic sciences, anthropology, and public health, revealing precious insight into the cultures and civilizations of past and present. This editorial is part of an article series published by the Academy for Multidisciplinary Neurotraumatology (AMN) https://www.brain-amn.org, showcasing some of the most important advancements, striking figures, and fascinating stories in neurotrauma, starting with the prehistoric age.

The umbrella term *neurotrauma* includes Acquired Brain Injury (ABI) and Spinal Cord Injury (SCI). While injuries to the brain can cause many problems, including troubles with attention, planning, and interaction, the primary result of spinal cord injury remains paralysis [1]. Traumatic Brain Injury (TBI) is an injury caused by a shock to the head or body and can result from falls, vehicle collisions, sports injuries, explosive blasts, combat injuries, gunshot wounds, or assault [2]. Some risk factors include age under 24 or over 60 and male gender. Depending on the severity, neurotrauma may result in temporary effects on the neuronal tissue or long-term damage, often due to bruising, bleeding, or torn tissue [2]. Moreover, brain injuries can cause mental health issues, including depression and behavioral dysfunction [1]. The advancement in the treatment of neurotrauma over the years has been tremendous, yet there is so much we have yet to know about the mechanisms and factors associated with it.

Mentions of TBI appear far back in the story of humanity, starting in myths and legends [3, 4]. Before scripts have been documented, archeology has been the main source of information, revealing the first insight into the intricacies of neurotrauma [5]. Prehistory included three main periods: Paleolithic, Neolithic, and Metal Age, with the ancestors of humankind (*Homo Habilis, Australopithecus, Homo erectus*) as well as *Homo sapiens* being traced back to the Paleolithic, when most of the head trauma was caused by animals or injuries [5]. One of the first pieces of evidence of brain injuries in humans was found in Tanzania, where the injury appears to have been caused by a crocodile bite [5]. However, the first anthropological signs of TBI treatment are attributed to the Neolithic era [5–7]. Researchers discovered signs of the ancient procedure of the *trephination*-*the oldest documented surgical procedure performed by humans*-on over 1500 skulls (5–10% of all found skulls) worldwide (Figure 1) [5, 8].

**Figure 1.**
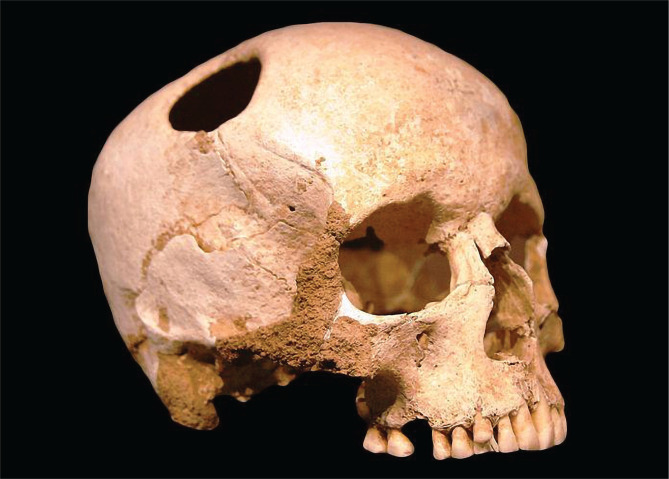
Skull with Trephination.

Interestingly, the practice is still used to treat chronic subdural hematoma (SDH) [7]. Trephination has been described as the process of “*scraping, cutting, or drilling of an opening (or openings) into the neurocranium”*, with most procedures including one or two holes [9]. However, there are documented cases with up to eleven holes [7]. Some of the skulls found presented signs of healing, indicating the patient’s survival post-procedure [7, 8, 10]. The origin of this practice and its widespread use is hypothesized to have begun in the pursuit of explaining the momentary loss of consciousness post-injury and the trial of “awakening” essential members of the tribe by “opening a gate” to facilitate the passage of spirits through the head [5, 8, 10]. The practice might have also been used as an initiation rite or ritual or to treat various disorders (headache, epilepsy, hydrocephalus, mental disorders). Surprisingly, there were high survival rates for the procedure, up to 70% in Inca procedures and Papua New Guinea [7]. Although trephination reached a peak in the Andes of ancient Peru (500 B.C.–500 A.D.), many ancient cultures from all over the world (including Europe, Asia, Africa, America) have experimented with trephination, with the practice being continued to modern days, in regions from Africa, South America, and Melanesia [5, 7, 9, 10]. Historically, the practice continued worldwide until the medieval period [7]. Sometimes, postmortem trepanations (the openings created by the procedure) [11] were used as a study method to gain insight and knowledge of the human body [7].

The data from Neolithic reveals insightful patterns in neurotrauma, including equal affection of men and women, the rarity of sharp-force trauma (suggesting intra-group violence and accidents as causes, rather than inter-group violence), a higher frequency of antemortem trauma in earlier periods, increasing size of the lesion with time, as well as a more common occurrence of healed lesions in the northern Mesopotamia [12]. The discrepancy in prevalence is hypothesized to root in early state formation leading to decreased violent conflicts. Moreover, the lower frequency of cranial trauma in the later times is generally accompanied by the larger average size of the lesion, suggesting a transition towards stone missiles and sling stones [12]. In most adult cases, antemortem cranial trauma was attributed to interpersonal violence and stands as the most reliable indicator of violence in anthropological research [12].

In ancient Egypt, one of the most outstanding progress in the field of neurotrauma research has been documented in the Edwin Smith Papyrus (1600 B.C.), *the first written documentation of brain injuries, the first known surgical treatise, as well as the first medical document in history* (Figure 2) [3, 5, 13]. It portrays the understanding of neurotrauma, as well as of other ailments, with mentions of the dura, cerebrospinal fluid, skull fractures with ipsilateral eye deviation and hemiparesis, temporal wound accompanied by speech disturbance, along with the first mention of aphasia due to cranial injury [3, 13].

**Figure 2.**
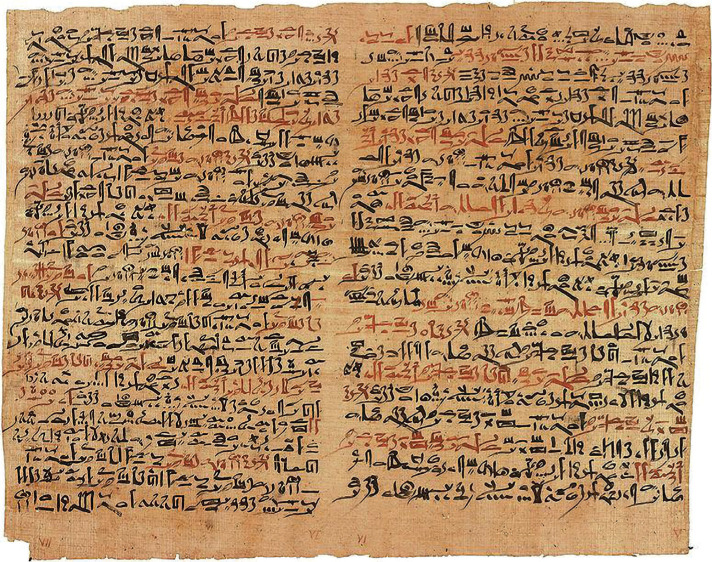
The Edwin Smith papyrus, the world’s oldest surviving surgical document. Written in hieratic script in ancient Egypt around 1600 B.C., the text describes anatomical observations and the examination, diagnosis, treatment, and prognosis of 48 type.

The papyrus also showcased the symptoms and the diagnostic procedure in patients with TBI, along with the therapeutic principles and prognostic detailed in specific cases, including mentions of spinal cord injuries with categorization [13, 14]. Among treatable wounds, the papyrus described “wounds without penetration of the skull or combined with a circumscription perforation of the skull, a putative impression, and meningism due to traumatic subarachnoid hemorrhage or meningitis” [13].

Treatments included applying fresh meat, honey, or grease on the wound, dressing the wound for those without skull fractures, and positioning the patients upright. The cases’ presentation is well-structured and follows a logical order, representing a possible guideline for the management of combating head injuries [13]. The papyrus gives insight into the high incidence of TBI at that point in time, showcasing the fantastic observational skills of the asû (physician), who had responsibility for physical treatments [5, 15]. The papyrus is based on an examination, logical analysis, and deductive reasoning, and it portrays 48 cases of trauma by anatomic region, 27 of which are head injuries [5, 16].

The document mentions four sections (title, examination, diagnosis, treatment) and a classification of fractures into 1) splits, 2) smashes, 3) compound comminuted, and 4) comminuted and depressed [16]. It was named after the American Egyptologist who obtained the papyrus. It is believed that the Greeks, including Hippocrates, knew the papyrus, as the observations presented in the papyrus were reintroduced during Greek times. However, the teachings of this historical document were furthermore disregarded until after the Dark Ages [16]. The papyrus also documents the ancient practice of trephination, discussed in the first article of the series. Historically, head trauma has been a good indicator of violence, with anthropological evidence revealing a decrease in violence with the establishment of states and professional armies, progresses which made farmers and city dwellers less prone to involvement in violent conflicts [15, 18, 19].

While neurotramatology has made significant progress throughout the centuries, its history provides a fascinating insight into the medical landscape of our ancestors. The broad gaps between our eras highlight how humanity has exploited the understanding gained over time to kindle and forge scientific and technological progress – modern neurotraumatology is certainly *standing on the shoulders of giants!*

## References

[1] What is Neurotrauma?. (n.d.). Ontario Neurotrauma Foundation.. https://onf.org/about/what-is-neurotrauma/.

[2] Traumatic brain injury - Symptoms and causes - Mayo Clinic. (n.d.). https://www.mayoclinic.org/diseases-conditions/traumatic-brain-injury/symptoms-causes/syc-20378557.

[3] McCrory PR, Berkovic SF (2001). Concussion: the history of clinical and pathophysiological concepts and misconceptions.. Neurology.

[4] Iaccarino MA, Bhatnagar S, Zafonte R (2015). Rehabilitation after traumatic brain injury.. Handb Clin Neurol.

[5] Bertullo G (2015). History of Traumatic Brain Injury (TBI).. American Journal of BioMedicine.

[6] Granacher RP Traumatic Brain Injury: Methods for Clinical and Forensic Neuropsychiatric Assessment, Second Edition..

[7] Lee KS (2015). History of Chronic Subdural Hematoma.. Korean J Neurotrauma.

[8] Faria MA (2015). Neolithic trepanation decoded - A unifying hypothesis: Has the mystery as to why primitive surgeons performed cranial surgery been solved?. Surg Neurol Int..

[9] Verano JW (2017). Reprint of-Differential diagnosis: Trepanation.. Int J Paleopathol.

[10] Stein SC, Georgoff P, Meghan S, Mizra K, Sonnad SS (2010). 150 years of treating severe traumatic brain injury: a systematic review of progress in mortality.. J Neurotrauma.

[11] Lewis M, Lewis M (2018). Chapter 5 - Trauma and Treatment With contributions from Petra Verlinden.. Paleopathology of Children..

[12] Sołtysiak A (2017). Antemortem Cranial Trauma in Ancient Mesopotamia.. International Journal of Osteoarchaeology.

[13] Kamp MA, Tahsim-Oglou Y, Steiger HJ, Hänggi D (2012). Traumatic brain injuries in the ancient Egypt: insights from the Edwin Smith Papyrus.. J Neurol Surg A Cent Eur Neurosurg.

[14] van Middendorp JJ, Sanchez GM, Burridge AL (2010). The Edwin Smith papyrus: a clinical reappraisal of the oldest known document on spinal injuries.. Eur Spine J.

[15] Reynolds EH, Wilson JV (2014). Neurology and psychiatry in Babylon.. Brain.

[16] Stiefel M, Shaner A, Schaefer SD (2006). The Edwin Smith Papyrus: the birth of analytical thinking in medicine and otolaryngology.. Laryngoscope.

[17] Panourias IG, Skiadas PK, Sakas DE, Marketos SG (2005). Hippocrates: a pioneer in the treatment of head injuries.. Neurosurgery.

[18] Sołtysiak A. (2017). Antemortem Cranial Trauma in Ancient Mesopotamia.. International Journal of Osteoarchaeology.

[19] ANE TODAY - 201602 - Head Injuries in Ancient Mesopotamia: What do we Really Know? - American Society of Overseas Research (ASOR) (n.d.). https://www.asor.org/anetoday/2016/02/head-injuries-in-ancient-mesopotamia-what-do-we-really-know/.

